# Oral mucositis management in hematopoietic cell transplantation in Australia and New Zealand (CAN EAT Survey)

**DOI:** 10.1007/s00520-026-10423-5

**Published:** 2026-03-03

**Authors:** Emma Munro, Nicole C Gavin, Midori Nakagaki

**Affiliations:** 1 Cancer Care Services, Royal Brisbane and Women’s Hospital, Brisbane, Australia; 2 Nursing and Midwifery Research Centre, Royal Brisbane and Women’s Hospital, Brisbane, Australia; 3 School of Nursing, Queensland University of Technology, Brisbane, Australia; 4 The University of Queensland, UQ Centre for Clinical Research, Brisbane, Australia; 5 Pharmacy Department, Royal Brisbane and Women’s Hospital, Brisbane, Australia; 6 Herston Infectious Disease Institute, Brisbane, Australia

**Keywords:** Oral mucositis, Assessment, Prevention, Treatment, Survey

## Abstract

**Purpose:**

The Multinational Association of Supportive Care in Cancer and the International Society of Oral Oncology (MASCC/ISOO) has published evidence-based international guidelines for the management of oral mucositis (OM). However, adherence to these guidelines has not been evaluated. This survey assessed OM management in hematopoietic cell transplantation (HCT) centers in Australia and New Zealand.

**Methods:**

Specialist nurses in 41 transplant hospitals were invited to complete a survey. Questions covered general unit information, oral assessment (tools, assessors, frequency), prevention strategies (guidelines, oral care, cryotherapy, photobiomodulation, palifermin, and barriers to use), and treatment strategies (pain management and nutritional support).

**Results:**

Of 41 hospitals, 24 (59%) responded. All but one responder were specialized nurses. Oral assessment was primarily conducted by nurses (100%) and doctors (80%). The most commonly used tools were the World Health Organization grading scale (42%), pain scores (46%), and EviQ (Australian online cancer resource) (42%). EviQ guidelines (67%), which exclude palifermin and photobiomodulation, followed by institutional guidelines (29%) and MASCC/ISOO guidelines (17%). Oral care (100%) and cryotherapy with melphalan (96%) were widely adopted, while photobiomodulation and palifermin were rarely used due to cost and access barriers. For OM-related pain, as-needed opioids (100%), patient-controlled analgesia (58%), and topical analgesics (46%) were common. When OM impaired oral intake, supportive measures included supplemental drinks (88%), enteral nutrition (50%), and parenteral nutrition (67%).

**Conclusion:**

In Australia and New Zealand, national EviQ guidelines are used more often than the MASCC/ISOO guidelines. Feasibility for implementing palifermin and photobiomodulation should be reassessed, and greater collaboration in developing guidelines is necessary.

## Background

Hematopoietic cell transplantation (HCT) is a life-saving treatment for patients with high-risk or relapsed hematologic malignancies. It involves the infusion of hematopoietic stem cells, either autologous or allogeneic, following intensive conditioning regimens that include high-dose chemotherapy and/or total body irradiation (TBI), or less toxic conditioning regimens [1, 2]. Oral mucositis (OM) is a debilitating complication of conditioning that can significantly impair quality of life and increase infection risk [3–5].

To address this, the Multinational Association of Supportive Care in Cancer and International Society of Oral Oncology (MASCC/ISOO) has developed clinical practice guidelines based on extensive published evidence for the prevention and treatment of OM [6]. For HCT patients, MASCC/ISOO recommends a combination of strategies, including basic oral care, cryotherapy with high-dose melphalan in autologous HCT, palifermin with TBI-based autologous HCT, and photobiomodulation (PBM) [6]. While basic oral care, such as bland mouth rinses, patient education, and dental professional care, lacks strong evidence, the panel reached consensus to recommend this practice as it is potentially beneficial for patient outcomes. Cryotherapy mitigates OM severity by reducing melphalan distribution in the oral mucosa [6]. Intraoral PBM has shown promising results in published studies, although it requires specialized equipment and training. In addition, palifermin, a keratinocyte growth factor, is recommended for patients undergoing TBI-based conditioning with autologous HCT, as it has been shown to reduce the incidence and severity of OM [6].

Although MASCC/ISOO provides evidence-based international guidelines, a number of national, regional, and institutional guidelines for OM management are currently in use. For example, the UK Oral Management in Cancer Care Group (UKOMiC) developed recommendations based on expert consensus as well as evidence-based guidance, emphasizing extensive oral care tailored to risk factors [7]. In Australia, the web-based EviQ guidelines are widely used [8]. While adopting MASCC/ISOO recommendations, EviQ modifies them to the Australian context, excluding palifermin and PBM due to limited access. As a result, basic oral care, patient-controlled analgesia (PCA), and cryotherapy remain the primary recommendations for HCT patients. Despite the availability of evidence-based MASCC/ISOO guidelines, clinicians continue to face challenges in OM management, with palifermin remaining the only pharmacologic intervention proven to significantly reduce OM incidence in HCT patients. Anecdotally, centers employ a wide range of preventive and therapeutic strategies, underscoring the lack of a standardized approach in this population.

Guidance on OM assessment has also been limited. Until the recent MASCC/ISOO publication [9], no formal recommendations existed. Commonly used OM assessment tools include the World Health Organization (WHO) scale and the Common Terminology Criteria for Adverse Events (CTCAE) [10, 11], both now are recommended by MASCC/ISOO for clinical practice and research. MASCC/ISOO also recommends incorporating patient-reported outcome measures (PROMs). Prior to the MASCC/ISOO publication, EviQ changed their recommendation from Oral Assessment Guide (OAG) [12] to CTCAE. Table 1 summarizes MASCC/ISOO and EviQ recommendation in HCT patients.

**Table 1:** MASCC/ISOO and EviQ recommendations/suggestions on OM management in HCT patients (clinical setting)

	MASCC/ISOO	EviQ
Assessment	WHO*, PROMs** (2024)	OAG*** à CTCAE^†^
Prevention	Basic oral care (including education)	Basic oral care
	Cryotherapy in autologous patients using melphalan	Cryotherapy in patients using melphalan
	PBM^††^	----
	Palifermin in autologous patients with TBI^†††^ based conditioning	----
Treatment	PCA morphine	PCA morphine

*WHO, world health organization; **PROMs, patient reported outcome measures; ***OAG, oral assessment guide;*CTCAE*, common terminology criteria for adverse events; ^††^*PBM*, photobiomodulation; ^†††^*TBI*, total body irradiation; *PCA*, patient-controlled analgesia

Despite the availability of guidelines, their real-world application and adherence across HCT centers have not been systematically evaluated. A clear understanding of current practices is crucial to improving OM management, ensuring that patients receive optimal care, and guiding future research into more effective therapies and guideline development. This survey therefore aimed to assess strategies used by HCT centers in Australia and New Zealand for OM assessment, prevention, and treatment. Identifying variations in practice and evaluating adherence to MASCC/ISOO recommendations provides valuable insights into the current state of OM management and can inform future efforts to standardize care and improve outcomes for HCT patients.

## Methods

This study was a survey of institutional practices for the management of OM across HCT centers in Australia and New Zealand. The project was approved by the Royal Brisbane and Women’s Hospital Human Research Ethics Committee (HREC/2022/RBWH/85181). This study was conducted in accordance with the Declaration of Helsinki. Participants were recruited via email and received an information sheet detailing the purpose of the survey, procedures for data management and confidentiality, and their rights regarding participation and withdrawal. Participation was voluntary, with consent implied upon survey completion.

All centers performing autologous or allogeneic HCT in adults were identified through the Australian BMT Recipient Registry and/or the Hematology Society of Australia and New Zealand. Specialist nurses, nurse unit managers, or BMT coordinators were contacted by e-mail and invited to participate. The survey (Appendix 1) included questions on responder and center characteristics (role of the responder, number of autologous/allogeneic HCTs performed), OM assessment (tools, frequency, assessors), prevention strategies (guidelines followed, specific interventions used, and barriers to use), and treatment approaches (pain management and nutritional support). The survey was conducted from March 2022 to February 2023. Descriptive analyses were performed to address the research objectives. The frequency of practices across hospitals was summarized as percentages. Free-text responses were grouped where applicable and reported as counts or percentages.

## Results

### Participants

A total of 41 centers were invited to participate in the survey, and 24 sites (22 in Australia and 2 in New Zealand) completed it, yielding a response rate of 59%. Respondents were predominantly specialist nurses, with one dietitian also participating. Allogeneic HCT was conducted in 12 of 24 centers (50%), while all but one center performed autologous HCT.

### Oral assessment

Table 2 summarizes the OM assessment tools used across centers. A variety of tools were employed, with the WHO assessment scale (42%), EviQ (42%), and pain scores (46%) being the most common. During the study period, EviQ recommendations for OM assessment were updated, shifting from the OAG to an adaptation of the CTCAE. Several centers reported using site-specific assessment tools. Oral assessments were performed by nurses in all centers (24; 100%) and additionally by doctors in 19 (79%) centers. Only 4 centers (17%) reported patient self-assessment. Assessments were conducted daily or more frequently in most centers.

**Table 2:** Oral assessment tools used in centers in Australia and New Zealand

Assessment Tools	Number of hospitals (N=24)	Brief explanation
WHO*	10 (42%)	Graded based on pain, oral ulcers and oral intake (1979)
Pain Score	11(46%)	Patient reported pain score or Visual Analogue Scale (VAS)
EviQ(either OAG or CTCAE)	10 (42%)	National recommendation – changed from Oral Assessment Guide (OAG) (Eilers 1988) to CTCAE during the surveyOAG: total score of various oral changes
CTCAE**	3 (13%)	Severity of symptoms and intervention needs
Institutional	4 (17%)	Other institutional
Other tools	2 (8%)	
No assessment tools	1 (4%)	

*WHO: world health organization, **CTCAE: common terminology criteria for adverse events

### OM prevention strategies

Hospitals followed different guidelines for OM prevention: 16 (67%) used EviQ, 4 (17%) used MASCC/ISOO guidelines, 7 (29%) used institutional guidelines, and 1 reported not following any guideline. Table 3 details the prevention strategies used and the reported reasons for not adopting specific interventions. The most frequently implemented strategies were cryotherapy with melphalan (23/24, 96%) and supportive oral care (24/24, 100%). Common basic oral care interventions included sodium bicarbonate (21/24, 88%), normal saline (12/24, 50%), and patient education (16/24, 67%). Palifermin was used in only 3 centers, restricted to patients undergoing TBI-based regimens, with cost and access cited as primary barriers. PBM was reported by only 1 center, also limited by accessibility. Awareness of PBM was generally low.

**Table 3:** OM prevention used in the centers and the reasons not using

Intervention	Number of centers (%) *N* = 24	Reasons not using				
		No access	cost	No knowledge	No evidence	Other
Palifermin with TBI*	3 (13%)	4	4	1	4	7 (No TBI)
PBM**	1 (4%)	12	1	10	1	
Cryotherapy	23 (96%)					1 (theoretical risk)
Oral care	24 (100%)					

*TBI, total body irradiation; **PBM, photobiomodulation

### Pain and nutrition management

**Fig. 1:**
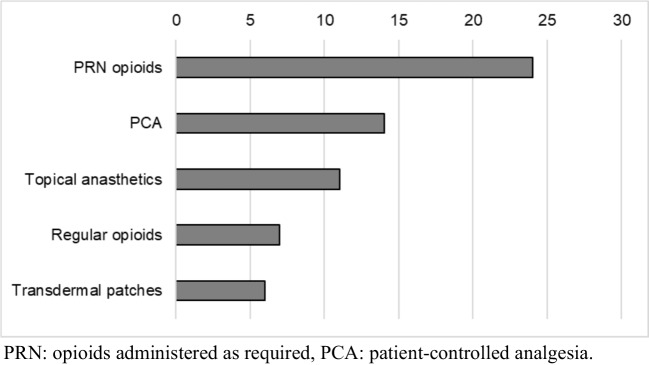
Pain management strategy (*N* = 24)

**Fig. 2:**
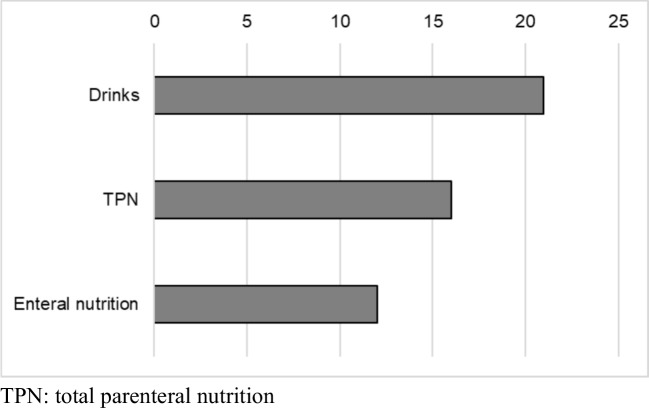
Nutrition supplement (*N* = 24)

There are no established treatments for OM. Management strategies focus on pain control and nutritional support. Figures 1 and 2 illustrate the pain management and nutritional supplementation methods used across centers.

## Discussion

This survey found that adherence to MASCC/ISOO guidelines was limited in Australia and New Zealand compared with the national EviQ guidelines, largely due to access issues and lack of awareness. However, all contents of EviQ guidelines are derived from MASCC/ISOO guidelines. Clinical practice, including assessment, prevention, and treatment of OM, was inconsistent across centers. To our knowledge, this is the first survey conducted in Australia and New Zealand, where national guidelines exist but MASCC/ISOO-recommended interventions remain difficult to access.

The WHO scale was the most commonly used oral assessment tool by clinicians. Pain scores were also frequently used, often in combination with other tools. The recently published new MASCC/ISOO guidelines on OM assessment, recommending daily use of the WHO scale, were published after this survey was completed [9] and therefore did not influence these results. Nonetheless, clinical practice in Australia and New Zealand was relatively aligned with the new recommendations regarding clinician-led assessments. In contrast, no centers reported routine use of PROMs apart from pain scores, a finding similar to the MASCC/ISOO survey [9]. MASCC/ISOO now recommends validated PROMs such as OMWQ [13], OMDQ [14], and PRO-CTCAE [15]. PROMs can capture subjective mucositis symptoms, including throat mucositis, which has been reported to be more common than OM [16], and can complement the limitations of the WHO scale, which focuses primarily on oral ulceration. The use of PROMs on a daily basis would enhance patient involvement in the assessment and management of OM, which may result in better clinical outcomes. This survey also found that nurses and doctors often conducted separate OM assessments. While multidisciplinary assessment may enhance accuracy, frequent or duplicate assessments risk placing an additional burden on patients. Greater use of PROMs and direct patient involvement in assessment may reduce this burden and improve accuracy.

Prevention strategies were largely limited to cryotherapy with high-dose melphalan and basic oral care, reflecting EviQ recommendations [8]. These measures are widely used because of their accessibility, low cost, and minimal adverse effects, although basic oral care is recommended mainly based on expert opinion [6]. Palifermin, a keratinocyte growth factor approved by the Food and Drug Administration (FDA), is not approved by the Therapeutic Goods Administration (TGA) in Australia. MASCC/ISOO recommends palifermin only in autologous HCT with TBI-based conditioning, based on a well-designed randomized trial in patients receiving TBI–etoposide–cyclophosphamide conditioning [17], a regimen associated with extremely high OM risk (grade 3–4 OM in 98% of placebo patients). However, subsequent trials in other HCT populations have shown conflicting results [18–22]. Overall, it appears that palifermin is effective only to prevent TBI-associated OM. Limited number of institutions that use TBI, high cost of the drug, and requirement for institutional approval for importing and paying for this unapproved drug can all contribute as barriers to the implementation of palifermin. PBM has demonstrated more consistent benefits across a wider range of HCT patients [23–30]. While PBM is a medical device and several products are approved by TGA, there are no national-level qualifications and training to be able to provide PBM, and this is regulated by each state government. Some states require training under already experienced PBM providers, but it may be difficult for first-time users in the field. It is essential that institutions fund a designated position responsible for providing PBM. Overall, it is possible to implement PBM at least in Australia, and appropriate funding and initiative would enable this. Participants in this survey cited both economic and accessibility barriers to the implementation of palifermin and PBM. Nonetheless, a small number of centers managed to introduce them, suggesting feasibility. Health economic studies indicate that initial costs for palifermin and PBM may be offset by reduced hospital stays and supportive care needs [31, 32]. Dissemination of evidence and inter-center collaboration may facilitate broader implementation. Notably, the lack of awareness about PBM was evident among respondents, which may reflect clinical role, training level, or scope of practice. Greater engagement with international advisory bodies such as MASCC/ISOO, national organizations such as EviQ, and local educational initiatives may improve knowledge and uptake of evidence-based practices.

Pain management and nutritional support remain central to OM treatment. MASCC/ISOO currently recommends morphine PCA and has withdrawn support for fentanyl transdermal patches [6]. Delays in national and local guideline updates may explain the ongoing use of less optimal strategies, including transdermal patches. In this survey, PRN opioids and topical analgesics were also commonly reported. Pain management remains highly subjective, with limited evidence in HCT settings, and is often influenced by patient preferences, institutional protocols, and specialist availability. Given the acute, complex, and self-limiting nature of OM-related pain, this area may benefit from consensus-driven local guidelines. Optimal nutritional support is also critical in HCT patients. Our survey showed that PN and EN were both commonly used, with all centers also providing nutritional supplement drinks. However, practices varied, and evidence comparing EN and PN remains inconclusive. Recent studies suggest EN may offer advantages, including shorter hospital stays, fewer infections, improved survival, and reduced incidence of acute GVHD compared with PN [33, 34]. Supportive measures such as effective pain control and early nutritional intervention are essential for improving clinical and psychological outcomes in HCT patients. Although MASCC/ISOO does not currently recommend specific nutritional interventions, future guidelines may evolve as new evidence emerges.

This survey was limited by its small sample size and the predominance of nurse respondents, who may not have knowledge of international guidelines and each intervention, especially palifermin and PBM. Responses might have differed if other healthcare professionals had been more widely represented. However, the main purpose of the study was to identify institutional policies and practices rather than individual clinical practice. While institutional practices are probably captured in the survey, barriers for implementation may have affected nurse’s specific awareness. In addition, the dental profession was not involved either in design or response to the survey. In the institution where this survey was designed, dentists’ involvement in OM management is limited to pre-HCT assessment. From the survey results, the role of the dental profession was not mentioned in free text. This may be an Australia and New Zealand–specific issue compared to other countries with extensive dental involvement in OM management. Finally, it was a self-reported survey and possibly biased toward nursing perspectives and reflected personal knowledge. Despite these limitations, this study highlights several key needs: stronger patient involvement in assessment, more practical preventive interventions, enhanced collaboration in developing national and international guidelines, and improved dissemination of evidence-based international recommendations.

In conclusion, hospitals in Australia and New Zealand are relying on national guidelines EviQ, which is a modification of the MASCC/ISOO guidelines. Reassessing the feasibility of assessments and interventions currently not in use (PROMs, dental involvement, PBM, Palifermin) is necessary, alongside enhanced collaboration in developing national and international guidelines, and improved dissemination of evidence-based international recommendations. This is critical in improving patient outcomes and optimizing supportive care standards.

## Data Availability

The data generated within this study can be made available by reasonable request to the corresponding author.

## Appendix 1.

**Table 4.** Survey questionnaire

	**Questions**	
**Part-1: Participant information**		
	Hospital Name (optional)	
	Name of the person answering	
	Which country is your hospital in?	⎕Australia ⎕New Zealand
	Which state is your hospital in? (if AU)	⎕ACT ⎕NSW ⎕VIC ⎕QLD ⎕ SA ⎕WA ⎕ NT
1-1	Your professional stream?	⎕Medical ⎕Nursing ⎕Pharmacy ⎕Other (specify: )
1-2	Approximate number of allogeneic HSCT per year?	⎕Nil ⎕1–25 ⎕26–50 ⎕51–75 ⎕76–100 ⎕101-
1-3	Approximate number of autologous HSCT per year?	⎕Nil ⎕1–25 ⎕26–50 ⎕51–75 ⎕76–100 ⎕101-
1-4	Do you treat HSCT patients as outpatients?	⎕Yes (mostly) ⎕No (mostly) ⎕Mixture of both
**Part-2: Oral mucositis assessment**		
2-1	Who regularly assesses oral mucositis?	⎕Nurses ⎕Doctors ⎕Patients ⎕Other (specify: )
2-2	How often do you assess oral mucositis? (if inpatient)	⎕More than once a day ⎕Daily ⎕between daily and weekly ⎕Weekly ⎕Other (specify: )
2-3	Which assessment tools do you use? (please select all that apply)	⎕WHO ⎕CTCAE ⎕RTOG ⎕OMAS ⎕OAG / EviQ ⎕Hospital specific ⎕Pain score (VAS or rating) ⎕Other (specify: )
**Part-3: Prevention strategies**		
3-1	Do you follow any of the following guidelines If so which?	⎕MASCC ⎕EviQ ⎕Institutional ⎕Other (specify: )
3-2	Do you use oral care protocols?	⎕Yes ⎕No
3-3	If not using oral care protocols, why?	⎕No evidence ⎕No access ⎕ Cost ⎕ Safety concern ⎕Other (specify: )
3-4	What approaches do you use as oral care? (please select all that apply)	⎕N/S mouthwash ⎕Sodibic mouthwash ¨Other mouthwash ⎕Brushing technique ⎕ Education ⎕Other (specify: )
3-5	Do you use cryotherapy (sucking ice)?	⎕Yes, only with melphalan ⎕Yes, with melphalan and other chemotherapy ⎕No
3-6	If not using cryotherapy, why?	⎕No evidence ⎕we do not use melphalan ⎕No access ⎕ Cost ⎕ Safety concern ⎕Other (specify: )
3-7	Do you use photobiomodulation (low level laser therapy)?	⎕Yes, for all patients ⎕Yes, for selected patients ⎕No
3-8	If not using photobiomodulation/ laser, why?	⎕No evidence ⎕No access ⎕ Cost ⎕ Safety concern ⎕Other (specify: )
3-9	If using photobiomodulation, who administer?	⎕Nurses ⎕Doctors ⎕Dentists ⎕Other (specify )
3-10	Do you use palifermin?	⎕Yes, with TBI based autologous ⎕Yes, with TBI based allogeneic ⎕No ⎕Other (specify: )
3-11	If not using palifermin, why?	⎕No evidence ⎕we do not use TBI ⎕No access ⎕ Cost ⎕ Safety concern ⎕Other (specify: )
3-12	Do you use other prevention strategies? (please select all that apply)	⎕Caphosol ⎕Zinc supplement ⎕Benzydamine (Difflam) ⎕Glutamine ⎕Vitamin E ⎕Honey ⎕Nil
3-13	What other prevention strategies do you use?	Free text
**Part-4: Treatment strategies**		
4-1	What is the standard mucositis pain management? (please select all that apply)	⎕PRN opioids ⎕PCA ⎕Transdermal opioids ⎕Other (specify: )
4-2	What is the standard nutrient supplement?	⎕Drinks ⎕NG feed ⎕TPN ⎕Other (specify: )
4-3	What other treatment strategies do you use?	Free text
